# Statement of Retraction: Didox (A Novel Ribonucleotide Reductase Inhibitor) Overcomes bc.l-2 Mediated Radiation Resistance in Prostate Cancer Cell Line PC-3

**DOI:** 10.1080/15384047.2025.2607914

**Published:** 2025-12-21

We, the Editors and Publisher of *Cancer Biology & Therapy,* have retracted the following article:

Inayat, M. S., Chendil, D., Mohiuddin, M., Elford, H. L., Gallicchio, V. S., & Ahmed, M. M. (2002). Didox (A Novel Ribonucleotide Reductase Inhibitor) Overcomes bcl-2 Mediated Radiation Resistance in Prostate Cancer Cell Line PC-3. *Cancer Biology & Therapy*, *1*(5), 539–545. https://doi.org/10.4161/cbt.1.5.174

After publication, concerns have been raised by a third party regarding unexpected similarities in the image panels in Figure 6.

Further investigations conducted by the Journal and Publisher confirmed significant integrity concerns. When approached for an explanation, the authors have cooperated with the investigation, but have been unable to address the concerns raised.

As verifying the validity of published work is core to the integrity of the scholarly record, we are therefore retracting the article. The corresponding author listed in this publication has been informed.

We have been informed in our decision-making by our editorial policies and the COPE guidelines.

The retracted article will remain online to maintain the scholarly record, but it will be digitally watermarked on each page as “Retracted”.

